# Effects of grape seed extract on periodontal disease: an experimental study in rats

**DOI:** 10.1590/1678-77572016-0298

**Published:** 2017

**Authors:** Feyza Otan ÖZDEN, Elif Eser SAKALLIOĞLU, Umur SAKALLIOĞLU, Bülent AYAS, Züleyha ERİŞGİN

**Affiliations:** 1 Ondokuz Mayıs University, School of Dentistry, Department of Periodontology, Samsun, Turkey.; 2 Ondokuz Mayıs University, Faculty of Medicine, Department of Histology and Embriology, Samsun, Turkey.; 3 Giresun University, Faculty of Medicine, Department of Histology and Embriology, Giresun, Turkey.

**Keywords:** Grape seed extract, Periodontal diseases, Chronic periodontitis, IL-10, TGF-β

## Abstract

Natural compounds capable of modulating the host response have received considerable attention, and herbal products are suggested as adjunctive agents in periodontal disease treatment.

**Objective:**

This study aimed to demonstrate the effect of grape seed extract (GSE) on periodontitis.

**Material and Methods:**

Ligature induced periodontitis was created in 40 rats and they were assigned to four equal groups. One group was fed laboratory diet (group A) while three groups received GSE additionally. Silk ligatures were placed around the cervical area of the mandibular first molars for four weeks to induce periodontitis. The GSE groups were reallocated regarding GSE consumption as: for two weeks before ligation (group B; totally eight weeks), from ligation to two weeks after removal of the ligature (group C; totally six weeks), and for two weeks from ligature removal (group D; totally two weeks). Sections were assessed histologically and immunohistochemically. Inflammatory cell number (ICN), connective tissue attachment level (CAL), osteoclast density (OD), IL-10 and TGF-β stainings in gingival epithelium (GE), connective tissue (GC), and periodontal ligament (PL) were used as the study parameters.

**Results:**

Lower ICN, higher CAL, and lower OD were observed in the GSE groups (p<0.05). IL-10 was more intensive in the GSE groups and in the GEs (p<0.05). Group B showed the highest IL-10 for PL (p<0.05). TGF-ß was higher in the GEs of all groups (p<0.017).

**Conclusions:**

The results suggest anti-inflammatory activities of GSE, but further investigations are needed for clarification of these activities.

## Introduction

In response to periodontopathogenic microorganisms, pro-inflammatory cytokines are synthesized to induce and maintain the inflammatory response in periodontal disease. Concomitantly, anti-inflammatory cytokines are released to limit the duration and extent of the periodontal inflammatory process. Maintaining a balance between pro-inflammatory and anti-inflammatory cytokines is one of the manifestations of self-regulation in the body^8^. In certain individuals, an improper immune response may lead to overproduction of inflammatory mediators^13^. Thus, an imbalance between pro-inflammatory and anti-inflammatory response results in periodontal breakdown^24^. Differences in the individual host responses have led researchers to investigate miscellaneous host modulating therapies that may slow down the progression and severity of periodontal disease, and, also, may be protective against the disease^10^. Natural compounds capable of modulating the host response have received considerable attention for this purpose, and herbal products are suggested as adjunctive agents in periodontal disease treatment^4^ ^,^ ^11^.

Grape seed extract (GSE) has been proposed as a promising immunomodulator agent, particularly due to its proanthocyanidin (PA) content, and is a naturally occuring polyphenolic compound obtained from seeds of *Vitis vinifera*, which possesses a wide range of biological activities such as antioxidant, anticarcinogenic, and anti-inflammatory effects^2^ ^,^ ^18^ ^,^ ^22^ ^,^ ^29^. GSE may strongly inhibit osteoclast differentiation, reduce osteoclast activity, and stimulate bone formation through its positive action on osteoblast differentiation, and thus, may be beneficial for the treatment of inflammation associated with bone destruction^23^. GSE generates its anti-inflammatory effect by calibrating the delicate balance between pro-inflammatory and anti-inflammatory cytokines through regulating their release and gene expression^1^. However, there are limited data on the effects of GSE on healthy and diseased periodontal tissues. GSE was demonstrated to protect against collagen breakdown^16^, and had a bacteriostatic effect on the anaerobes that may significantly decrease the maturation of dental biofilm, and therefore, may be used in the prevention of periodontal disease^7^. Further studies are needed to particularly show the mechanisms of GSE interactions in periodontal inflammatory process.

Interleukin (IL)-10 is an anti-inflammatory cytokine that is produced by macrophages, T cells, B cells, mast cells, and keratinocytes for a major function of supressing cytokine and chemokine production from macrophages^5^. IL-10 mediates the control of periodontal disease progression^21^ and modulates the periodontal inflammatory response^26^. Transforming growth factor-beta (TGF-β) represents a family of polypeptide growth factors and plays an important role in tissue regeneration, remodeling, and fibrosis^19^, which has anti-inflammatory effects due to its roles in the suppression of collagenase production and in the enhancement of the tissue inhibitors of matrix metalloproteinases^6^. Gingival crevicular fluid (GCF) level of TGF-β has been found to be higher after periodontal treatment as a marker of inflammatory resolution and healing process^15^. Since the role of these cytokines are well-documented in periodontitis, the effect of GSE in periodontal inflammation was worth studying.

Since the microbial dental biofilm accumulation is the primary etiologic factor for periodontal disease, GSE may be useful in the presence of microbial dental biofilm induced periodontal inflammation, due to its anti-inflammatory action. The purpose of this study is to investigate the effects of GSE application on periodontium before and after ligature induced experimental periodontitis, using histological and immunohistochemical analyses.

## Material and Methods

All experimental procedures were approved by the Animal Ethics Committee and were performed according to the mandatory regulations of this Committe. Forty male Sprague Dawley rats with average weight of 150-200 g were used in the study. They were housed in an air-conditioned room at 23-25°C, exposed to a 24-h-light dark cycle of equal time, and fed standart laboratory diet and water *ad libitum*.

### Periodontitis induction and GSE application

Ligature induced experimental periodontitis were created in all animals. A 4/0 silk suture was placed in submarginal position around the right mandibular 1^st^ molar teeth and kept there for four weeks to induce periodontitis^12^. GSE was obtained from Berkem SA, Gardonne, France, and supplied in a form of standardised extract including +90% oligomeric PAs. It was daily prepared regarding each animal’s body weight and systemically administered via gavage feeding once a day. The dose of GSE was calibrated as 200 mg/kg BW according to a previous study^28^ and due to its proved antioxidant, anti-inflammatory, and antiapoptotic effect with the stated dosage^3^ ^,^ ^27^.

### Groups

**Figure 1:**
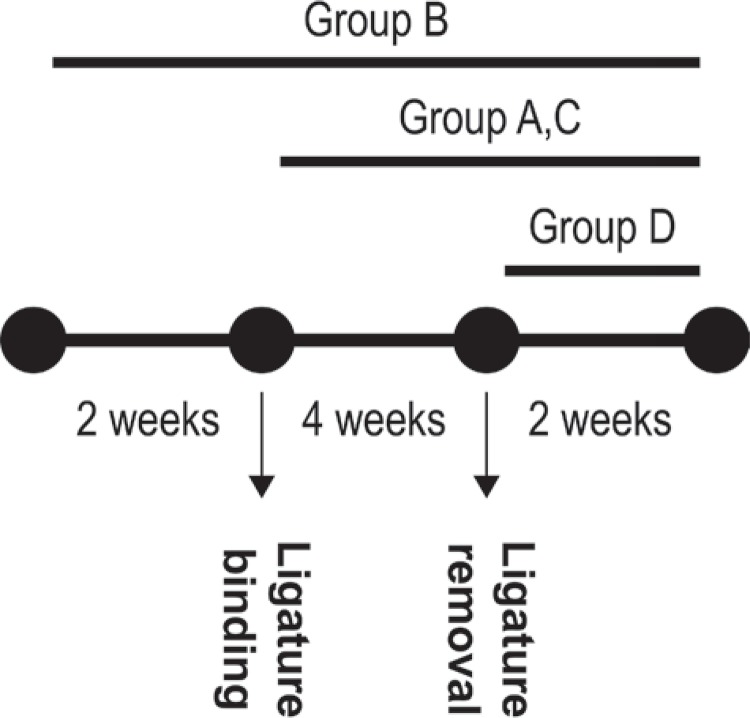
Designation of the experimental groups. Group A: control; Group B, C, and D: grape seed extract (GSE) applied groups

Animals were randomly divided into four groups (groups A, B, C, and D) with 10 rats in each one. Group A was used as a positive control group and the animals were fed only standard laboratory diet/water until two more weeks following ligature removal (totally six weeks). Group B included the rats that received GSE for two weeks before periodontitis induction and continued for six weeks (for a period of eight weeks). Group C included the rats that received GSE from the day of periodontitis induction and continued for six weeks (for a period of six weeks). Group D included the rats that received GSE after ligature removal and continued for two weeks (for a period of two weeks). Regarding the experimental periods, rats were sacrificed via overdose of anesthetic solution injection. The mandibles were removed, separated from muscle and soft tissues, and the right mandibular sides were used for histological and immunohistological assessments. Designation of the experimental groups is shown in Figure 1.

### Histological analysis

The right mandible was first fixed in neutral buffered formalin and decalcified with 10% formic acid solution. Followed by decalcification, the right mandible was embedded into paraffin and 5 µm-thick sections were made in a bucco-lingual direction throughout the first molar tooth. Each 40^th^ section was sampled in a systemic random sampling-manner and stained with hematoxylin eosin. Eight sections obtained in this way were used for histomorphometrical assessments that were performed by a camera-attached light microscope. Mesial site of the first molar tooth was selected for the assessments in each section. Inflammatory cell number (ICN), connective tissue attachment level (CAL), and osteoclast density (OD) was used as the parameters of histomorphometrical measurements. ICN was calculated in four different areas of 70x70 µm^2^ under the junctional epithelium. CAL was measured as the µm distance between alveolar crest and connective tissue border beneath the junctional epithelium^14^. OD was counted as the average numbers of four areas in 170x170 µm^2^ throughout the mesial alveolar bone.

### Immunohistochemical analysis

For each tooth, 2-3 sections in 5-µm thick were taken over poly-L-lysine coated slides for immunohistochemical analysis. Sections were deparaffinized with xylene, rehydrated through alcohol, and washed with distilled water. For antigenic unmasking, they were exposed to microwave radiation in a citrate buffer (pH: 6.0). Sections were then washed with distilled water, incubated in %3 H_2_O_2_, washed with phosphate-buffered saline (PBS; Ph: 7.2), and incubated with protein blocking solution for 10 minutes respectively (Super Block, SensiTek HRP Anti-Polyvalent Lab Pack, Scytek Laboratories, Logan, Utah, USA). IL-10 and TGF-β were used as the parameters of immunohistochemical assessments. Primary antibodies against IL-10 (0.1 mg, rabbit polyclonal antibody, Abbiotec, San Diego, CA, USA) and TGF-β (Anti-TGF-β antibody, Abcam, Cambridge, UK) were incubated for 60 minutes at room temperature. After washing with PBS, sections were exposed to biotinylated secondary antibody (Biotinylated Link Antibody, SensiTek HRP Anti-Polyvalent Lab Pack, Scytek Laboratories, Logan, Utah, USA) for 20 minutes. The sections were then washed with PBS, kept in streptavidin-conjugated peroxidase solution (Streptavidin/HRP Label, SensiTek HRP Anti-Polyvalent Lab Pack, Scytek Laboratories, Logan, Utah, USA) for 20 minutes, and exposed to diaminobenzidine (DAB: 3,3’-diaminobenzidine tetrahydro chloride, DBS, Pleasanton, CA, USA) for color development. Followed by washing with PBS, sections were counterstained with Mayer’s hematoxylin, dehydrated, and coated with Entellan^®^. Gingival epithelium (GE), gingival connective tissue (GC), and at 2/3 coronal part of mesial periodontal ligament (PL) were evaluated for their staining profiles of IL-10 and TGF-β. For the determination of staining intensity levels, immunoreactions against antibodies were quantified by scoring between 0-3 as: 0 (no immunoreactivity), 1 (light immunoreactivity), 2 (moderate immunoreactivity), and 3 (strong immunoreactivity)^25^. Two blinded examiners separately performed this scoring via light microscopy at x100 objective magnification and they determined the average scores of 10 different areas in GE, GC, and PL for each section. The HScore value was calculated for each tissue by adding percentage of the cells that stained at each staining intensity, and multiplying it by the weighted intensity of staining (HScore=ΣPi (i+1), i: intensity scores and Pi: percentage of the cells)^25^. Finally, the average HScore value of two examiners was used as one value in the calculations.

### Statistical analysis

Data analyses were performed using a statistical software program (SPSS Version 16.0, SPSS, Chicago, IL, USA). Power analysis showed a minimum allocation of 10 rats in each group to obtain the statistical significance at p<0.05 level with a power of 80%. The Shapiro-Wilk test was used to determine whether the data were normally distributed. Comparisons of the histomorphometrical profiles of the study groups were analyzed using the Kruskal-Wallis test. Differences between the groups for the immunohistochemical parameters in normal distribution were evaluated by One-way ANOVA test and homogenity of variances was calculated by Levene test, and the results were expressed as mean±standard deviation values. *Post hoc* Tukey test was performed to determine multiple comparisons among the compartments of the groups, and intragroup comparisons of GE, GC, and PL were made by repeated measures analysis of variance (*post hoc* paired sample test). Statistical significance level was accepted as p<0.05/3=0.017 for *post hoc* paired sample test and as p<0.05 for the other tests.

## Results

### Histomorphometrical assessments

**Figure 2:**
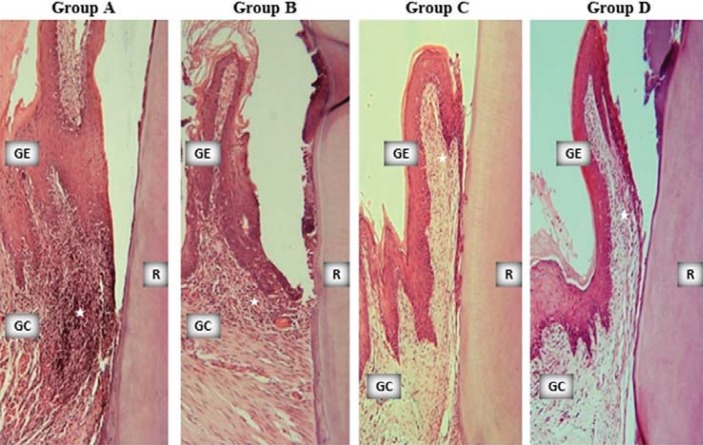
Sample microphotographs from the study groups (x10, hematoxylin-eosin). Stars indicate inflammatory cell infiltrate. GE: gingival epithelium, GC: gingival connective tissue, R: tooth root

ICN, CAL, and OD were shown in Table 1. All the groups showed signs of periodontal inflammation and tissue destruction in different degrees (Figure 2). However, ICN was lower in groups B, C, and D than in group A; CAL was higher in groups B, C, and D than in group A; and OD was lower in groups B, C, and D than in group A (p<0.05).

**Table 1:** Histomorphometrical profiles of the study groups. (ICN: Inflammatory Cell Number; CAL: Connective Tissue Attachment Level in µm; OD: Osteoclast Density)

Groups	ICN	CAL	OD
	Median (min-max)	Median (min-max)	Median (min-max)
A	9.83	525.00	0.06
	(4.5-14.66)	(50-1050)	(0.03-0.12)
B	6.99	825.00	0.04
	(6.00-19.83)	(425-1075)	(0.02-0.06)
C	5.83	875.00	0.04
	(2.16-19.50)	(410-1450)	(0.03-0.05)
D	6.5	825.00	0.03
	(4.83-20.83)	(490-1025)	(0.02-0.04)
p¥	0.032 *	0.012 *	0.039 *

¥: Kruskal Wallis test

*: Statistically significant (p<0.05)

### Intergroup comparisons of IL-10 and TGF-ß stainings

**Figure 3:**
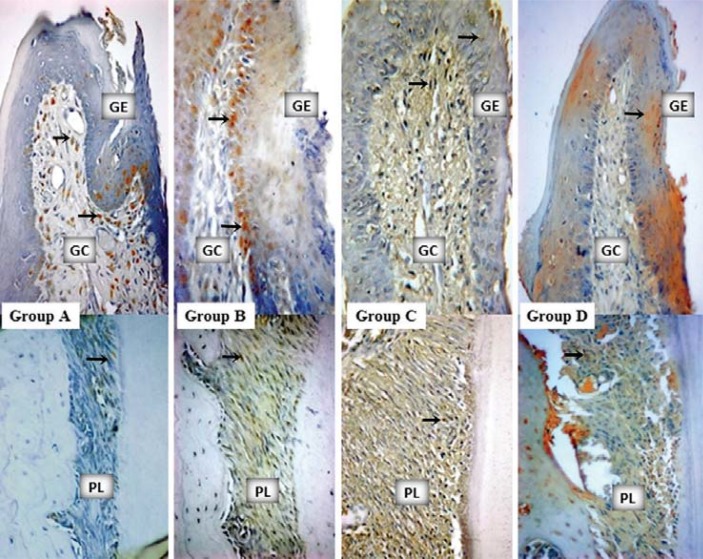
Mesial gingiva from the buccal–lingual sections of mandibular first molars. IL-10 staining samples from the study groups (x40, diaminobenzidine). Arrows indicate the stained cells. GE: gingival epithelium, GC: gingival connective tissue, PL: periodontal ligament

**Figure 4:**
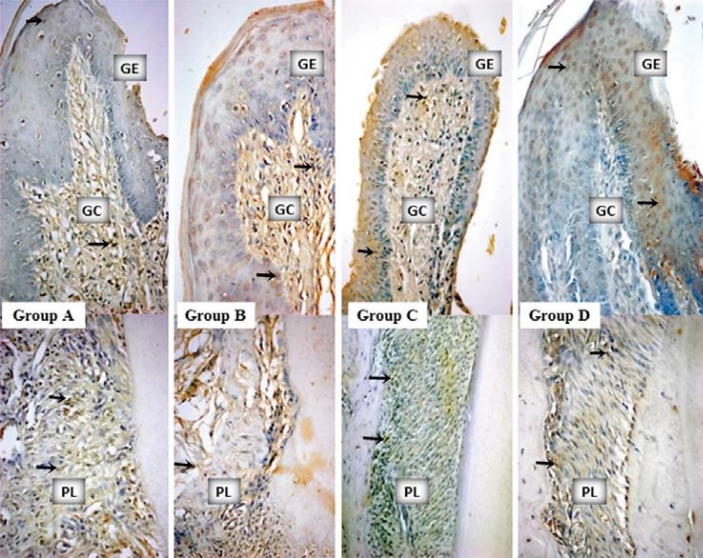
Mesial gingiva from the buccal–lingual sections of mandibular first molars. TGF-β staining samples from the study groups (x40, diaminobenzidine). Arrows indicate the stained cells. GE: gingival epithelium, GC: gingival connective tissue, PL: periodontal ligament

IL-10 staining profiles and intergroup comparisons as HScores were given in Table 2. More IL-10 accumulation was determined in the GEs of groups B, C, and D compared with the GE of group A (p<0.05) (Table 2, Figure 3). IL-10 stainings in GCs were similar for all the groups (p>0.05) (Table 2, Figure 3). Group B showed the most intensive IL-10 staining for PL (p<0.05). TGF-ß staining profiles and intergroup comparisons as HScores were given in Table 3. There were no statistically significant differences amongst the stainings of all the groups for GE, GC, and PL (Figure 4).

**Table 2:** HScore values with the inter- and intra-group comparisons of IL-10 staining. Data were expressed as as mean±standart deviation (GE: Gingival epithelium; GC: Gingival Connective Tissue; PL: Periodontal Ligament)

Groups	GE	GC	PL	p§
				F
A	157.21±37.64^a,b,c^	144.86±15.42	131.03±11.94^d^	0.000007*
				361.972
B	240.80±33.06^a^	183.72±29.13	195.56±40.11^d,e,f^	0.0000*
				
	p=0.000			418.716
C	227.84±36.59^b^	137.61±49.05	133.77±42.88^e^	0.00001*
				
	p=0.002			183.40
				
D	222.48±43.22^c^	155.38±38.39	138.86±41.25^f^	0.0000*
				
p¥	0.0004	0.081	0.002	
F	8.687	2.512	6.863	

¥: One-way ANOVA test

§: *Post hoc* Tukey test

a: Statistical difference between groups A and B (p<0.05); b: Statistical difference between groups A and C (p<0.05); c: Statistical difference between groups A and D (p<0.05); d: Statistical difference between groups B and A (p<0.05); e: Statistical difference between groups B and C (p<0.05); f: Statistical difference between Group B and Group D (p<0.05); *Statistical diference between GE, GC, and PL in the groups

**Table 3:** HScore values and comparisons of TGF-β staining. Data were expressed as mean±standart deviation (GE: Gingival epithelium; GC: Gingival Connective Tissue; PL: Periodontal Ligament)

Groups	GE	GC	PL	p^**b**^
				F
A	217.83±42.95	183.37±55.73	155.66±55.93	>0.05
				68.752
B	211.76±57.15	158.76±62.79	132.84±27.93	0.000186*
				96.556
C	207.45±45.46	159.42±46.45	141.36±31.92	0.000017*
				151.948
D	209.89±43.38	156.01±48.41	126.24±34.56	0.000001*
				861.700
Pa	0.981	0.764	0.513	
F	0.058	0.386	0.791	

One Way ANOVA test

^b^: *Post hoc* TUKEY test

*: Statistical differences amongst GE, GC, and PL in the groups

### Intragroup comparisons of IL-10 and TGF-ß stainings

Intragroup comparisons were summarized in Table 4. IL-10 staining did not show any differences between GE-GC and GE-PL in group A (p>0.017), but it was higher in GC than that of PL in this group (p<0.017). TGF-ß stainings were similar in all the compartments of group A (p>0.017). IL-10 staining was more intensive in GE compared with GC and PL in group B (p<0.017), and it was similar in the GC and PL of this group (p>0.017). TGF-ß was higher in the GE of group B than that of the PL in this group (p<0.017). IL-10 staining of GE was more intensive than GC and PL for groups C and D (p<0.017), but it was similar for the GCs and PLs of these groups (p>0.017). TGF-ß stainings of groups C and D were higher in GEs than those in PLs (p<0.017), and they were similar for the GEs-GCs and GCs-PLs of these groups (p>0.017).

**Table 4:** HScore values for intragroup comparisons of IL-10 and TGF-ß in the study groups (GE: Gingival epithelium; GC: Gingival Connective Tissue; PL: Periodontal Ligament)

Groups	Paired areas	IL-10	TGF-ß
		( p, ta)	( p, ta)
	GE-GC	0.406, 0.908	0.291, 1.179
A	GE-PL	0.115, 1.904	0.045, 2.651
	GC-PL	0.003, 5.491^b^	0.159, 1.655
	GE-GC	0.003, 4.490^b^	0.184, 1.540
B	GE-PL	0.005, 4.051^b^	0.005, 4.709^b^
	GC-PL	0.328, -1.052	0.076, 2.227
	GE-GC	0.001, 5.285^b^	0.025, 2.958
C	GE-PL	0.000, 17.080^b^	0.005, 4.266^b^
	GC-PL	0.670, 0.448	0.034, 2.739
	GE-GC	0.010, 3.580^b^	0.061, 2.404
D	GE-PL	0.000, 7.874^b^	0.002, 5.722^b^
	GC-PL	0.182, 1.480	0.092, 2.077

Paired Samples test

^b^: Statistically significant

## Discussion

The present investigation was designed to demonstrate the potential of oral GSE administration as a preventive or therapeutic agent for periodontal disease. We investigated anti-inflammatory effects of GSE on periodontal tissues by histomorphometrical and immunohistochemical means. Our findings proposed that GSE intake might have altered local inflammatory responses during the periodontal disease process.

Our histomorphometrical findings showed periodontal inflammation and destruction in all the groups in different severities by means of inflammatory cell infiltration, connective tissue attachment loss, and osteoclastic activity. The GSE usages that initiated prior to ligature placement (group B), on the day of ligature placement (group C), and on the day of ligature removal (group D) improved connective tissue level and bone healing, which was also associated with a lower degree of inflammation than the negative control group (group A). These findings may be supported by the results of some studies in which GSE decreased reactive oxygene and nitrogen species, inhibited myeloperoxidase and lysosomal enzymes activities in experimental periodontitis^9^, downregulated matrix metalloproteinases in inflamed periodontal tissues^16^, and inhibited osteoclast differentiation and reduced osteoclast activity in mice when applied as 100 mg/kg for 18 days^23^. Although the doses, methods, and periods of GSE application are dissimilar in these studies, all their findings show positive effects of GSE on bone formation and/or healing. Our OD results are also in agreement with this phenomenon as suggesting an inhibitory impact of GSE on osteoclastic activity.

Govindaraj, et al.^9^ (2010) were the first to demonstrate the anti-oxidant effect of proanthocyanidin in an experimental periodontitis model that was induced by injecting *E.coli* endotoxin. The same study consisted of different experimental periods (the longest was 30 days) and different dosages of proanthocyanidin (the highest was 40 mg/kg BW). Differently, our study showed the anti-inflammatory effect of GSE in ligature-induced experimental periodontitis for a longer duration (the longest was eight weeks) without any side-effects of a higher dosage (200 mg/kg BW).

The GSE has been reported to have an anti-inflammatory capacity due to its effect on oxidative stress, and therefore, it has been suggested as a remedy in the prevention of several inflammatory diseases^11^. Proanthocyanidins from grape seed inhibited tumour necrosis factor (TNF)-α and IL-1β formations in the exudate from edema paws of rats, thus suggesting its anti-inflammatory role as a suppressor of pro-inflammatory cytokines^18^. The present study results are in agreement with this finding, because we have determined the increased production of IL-10 due to GSE intake. In a recent study, the anti-inflammatory activity of GSE (orally administered as 25, 50, and 100 mg/kg doses) was explained with the decrease in Th1 and Th 17A levels and increase in Th2 released cytokines at the inflammation site^1^. This study also demonstrated that GSE increased the mRNA expressions of IL-10 and TGF-ß while reducing the IL-1ß and TNF-α levels^1^. Therefore, it may be proposed that GSE demonstrates its anti-inflammatory efficacy via regulating the releases of pro- and anti-inflammatory cytokines.

Our immunohistochemical findings suggested a possible anti-inflammatory effect of GSE. As the total GE area, more intensive IL-10 stainings were demonstrated in all the GSE groups compared with the non-GSE group. Group B, which was designed as the prophylactic group, had also the most abundant IL-10 cytokine staining in PL and in GC, although the last one was not found statistically different. Two weeks of GSE intake prior to experimental periodontitis in group B might have created such a difference in GE, GC, and PL due to its preventive effect on periodontitis. There was no preventive or anti-inflammatory effects after the disease was created and no effect to decrease the destruction (groups C and D) in GE, GC, and PL. IL-10 expression in the early phases of inflammation is coordinated with other genes’ expression that has potent pro-inflammatory and chemoattractant properties, but the functions of IL-10 converge into a congruent attempt at later inflammatory stages for limiting the damages of inflammation^20^. The stage-dependent property of this cytokine may explain the differences amongst the GSE groups in the presented study. The administration of GSE beginning two weeks before ligature placement (group B) presented the best results of IL-10 and the levels were higher in all compartments of group B. Although IL-10 was demonstrated to be higher in the GEs of other GSE groups (groups C and D), the differences were not significant in the connective tissue and the periodontal ligament when compared with the postive control group (group A). This means that the anti-inflammatory effect of GSE may be limited with the application time.

In the present study, there were no significant differences in the TGF-ß level amongst the groups. However, intragroup comparisons showed statistical differences in the selected regions. In all the GSE groups, TGF-ß staining was more intensive in GE compared with PL. This may be explained by the fact that gingival fibroblasts had almost a two-fold increase in the amount of TGF-β when compared with periodontal ligament fibroblasts^21^. There are no available data about the effect of GSE on TGF-β in gingival tissues. But it has been shown that GSE inhibits arsenic-related liver damage by TGF-β/Smad activation and suppression of NADPH oxidase^17^.

There is a growing interest in herbal remedies as adjunctive anti-inflammatory agents in the prevention of periodontal disease, particularly for the individuals prone to disease. This study evaluated the interaction between grape seed extract (GSE) and diseased periodontium in experimental peridontitis. Histopathological findings showed improvements in the inhibition of periodontal inflammation and destruction following GSE intake. Innmunohistochemical findings demonstrated more intensive IL-10 stainings in the GSE groups without significant differences in the TGF-ß levels.

## Conclusion

In conclusion, although our findings suggest an anti-inflammatory activity of GSE, further investigations are still needed to clarify possible mechanisms and details of this activity in the periodontal inflammatory process.
